# Supplementary material for: A novel performance scoring quantification framework for stress test set-ups
Source: PLoS One. 2023 Apr 27;18(4):e0284083. doi: 10.1371/journal.pone.0284083 (PMC10138280; doi:10.1371/journal.pone.0284083)
Supplement: S2 File — (PDF) [file pone.0284083.s002.pdf]

The full features list included in the real data example as extracted from completing the gait assessment. Each feature was calculated once for the usual walk and once for the dual-task walk

Total walking time
Number of strides during walk
Stride regularity
Gait asymmetry
Sample entropy
Step regularity during the straight line walking in TUG
Duration of the first turn
Number of strides without turns
Step regularity
Phase coordinator index
Total duration of the TUG
Step symmetry during the straight line walking in the TUG
Duration of the second turn (stand to sit)
CV (mean/std) of the step time, during the straight line walk, of the leg with the bigger mean step time
Stride time CV during the straight line walk
Step symmetry
